# Comparative Analysis of 24-Hour Post-Polymerisation Colour Change in Experimental Rice Husk Silica-Incorporated Flowable Composites with Different Photoinitiator Systems Across Three Colour-Assessment Devices

**DOI:** 10.3390/ma19173764

**Published:** 2026-09-04

**Authors:** Aqil Thaqif Rayyan Zaki, Eyas Abuhijleh, Mithaq R. Mohammed, Matheel AL-Rawas, Abdalbseet A. Fatalla, Tengku Fazrina Tengku Mohd Ariff, Johari Yap Abdullah, Mohd Firdaus Yhaya, Tahir Yusuf Noorani

**Affiliations:** 1 Biomaterials Unit, School of Dental Sciences, Universiti Sains Malaysia, Health Campus, Kubang Kerian, Kota Bharu 16150, Kelantan, Malaysia; 2 Department of Clinical Sciences, Center of Medical and Bio-Allied Health Sciences Research, College of Dentistry, Ajman University, Ajman 346, United Arab Emirates; 3 College of Dentistry, Al-Iraqia University, Baghdad 10053, Iraq; 4 Prosthodontic Unit, School of Dental Sciences, Universiti Sains Malaysia, Health Campus, Kubang Kerian, Kota Bharu 16150, Kelantan, Malaysia; 5 Department of Prosthodontics, College of Dentistry, University of Baghdad, Baghdad 61023, Iraq; 6 Faculty of Dentistry, Universiti Teknologi MARA, Sungai Buloh 47000, Selangor, Malaysia; 7 Craniofacial Imaging Laboratory, School of Dental Sciences, Universiti Sains Malaysia, Health Campus, Kubang Kerian, Kota Bharu 16150, Kelantan, Malaysia; 8 Conservative Dentistry Unit, School of Dental Sciences, Universiti Sains Malaysia, Health Campus, Kubang Kerian, Kota Bharu 16150, Kelantan, Malaysia; tahir@usm.my

**Keywords:** rice husk silica, flowable composite, photoinitiator, post-polymerisation colour change, CIELAB, device agreement

## Abstract

Background/Objectives: Rice husk silica (RHS) is a biosustainable filler with potential application in dental flowable composites (FCs). However, early post-polymerisation colour change in RHS-incorporated FCs may vary with material formulation, particularly resin composition and photoinitiator system, and may also be affected by the colour-assessment device used. This study compared the 24 h post-polymerisation colour change of 10 experimental RHS-incorporated FC groups formulated with five photoinitiator systems and two commercial FCs used as control, using three colour-assessment devices. Methods: Two UDMA/TEGDMA formulations (U50 and U40), each containing 50 wt% nanohybrid RHS, were prepared with different photoinitiator systems (CQ, PPD, CQ/PPD, BAPO, or TPO). CIELAB coordinates were recorded immediately after curing and after 24 h of dry, dark storage at 37 °C using D1 (VITA Easyshade), D2 (Ds-700d Portable Spectrophotometer), and D3 (ColorMeter Pro). The same specimens were measured with all three devices, with five matched specimens from each of the 12 composite groups (*N* = 60). A mixed repeated-measures ANOVA was performed with device as the within-subject factor and composite as the between-subject factor; agreement was additionally assessed using Bland–Altman analysis and an absolute-agreement intraclass correlation coefficient (ICC). Results: Device, composite, and the device × composite interaction were significant (all *p* < 0.001). Estimated mean ΔE*ab was highest for D1 (5.019), followed by D3 (2.368) and D2 (1.777), and all device pairs differed significantly after Bonferroni adjustment. Bland–Altman analysis showed systematic positive bias for D1 relative to D2 and D3. The absolute-agreement ICC was 0.130 (95% CI 0.078–0.177), indicating poor agreement. Conclusions: The magnitude of early 24 h colour change depended on both the composite material and the measurement device. The three devices should not be assumed to be directly interchangeable for ΔE*ab assessment; the same calibrated device should be used throughout a longitudinal comparison.

## 1. Introduction

Flowable composites (FCs) are widely used in restorative dentistry because they offer favourable handling, low viscosity, good cavity adaptation, and the ability to wet irregular surfaces. These characteristics support their use in small cavities, liners, repair procedures, minimally invasive restorations, cervical lesions, and other areas where adaptation is difficult using conventional paste composites [1,2]. However, the same compositional features that improve flowability may also affect long-term aesthetic behaviour. FCs generally contain a higher proportion of organic resin matrix and a lower filler loading than conventional packable composites; consequently, they may be more vulnerable to water sorption, solubility, surface degradation, and pigment uptake when exposed to the oral environment [1,3,4]. Because visible colour change is one of the main reasons for dissatisfaction and replacement of aesthetic restorations, colour stability remains an important criterion when developing or comparing flowable composite materials [5,6]. Importantly, FCs constitute a heterogeneous family rather than a single uniform material class. They may be differentiated according to viscosity and rheology, filler loading and morphology, resin composition, mechanical behaviour, curing design, and intended clinical indication. Clinically oriented categories include conventional low-viscosity flowables, self-adhering materials, bulk-fill flowables, core/build-up materials, and newer bioactive or repair-oriented formulations, with some overlap between categories [1]. Accordingly, findings from one formulation should not be generalized to all FCs.

Colour stability describes a restorative material’s ability to maintain its appearance over time. Discoloration may result from external staining or internal changes within the resin matrix, including residual amines, unreacted monomers, water sorption, and filler–matrix alterations [7,8,9]. Common beverages such as coffee, tea, cola, and red wine can further affect colour, with the extent of change depending on material composition, surface characteristics, exposure time, and measurement method [4,10,11]. From a clinical perspective, maintaining optical stability is essential for continued visual integration with surrounding tooth structure. Noticeable discoloration can produce restoration-tooth mismatch and marginal disharmony, reduce patient satisfaction, and prompt repolishing, repair, or premature replacement even when the restoration remains mechanically serviceable. Repeated replacement may also sacrifice additional tooth structure and shorten the restoration life cycle [5,6].

The resin matrix and filler system can influence the optical behaviour of resin composites. Water sorption, residual monomers, filler loading, particle characteristics, filler–matrix coupling, and refractive-index matching may all affect colour and translucency [4,6,12,13,14,15]. These factors are particularly relevant to experimental rice husk silica (RHS)-incorporated FCs, in which the filler source and its interaction with the resin matrix may differ from those of commercial materials.

Rice husk is an abundant agricultural by-product and a sustainable source of silica for dental-composite fillers. RHS-derived silica has been incorporated successfully into experimental FCs and evaluated for properties such as degree of conversion, water sorption, solubility, hardness, surface characteristics, and depth of cure [14,16,17]. These findings support RHS as a promising filler material while also highlighting the importance of resin formulation in determining its overall performance.

Photoinitiator chemistry may also influence the colour behaviour of light-cured composites. Camphorquinone (CQ), the most widely used dental photoinitiator, has an intrinsic yellow colour and commonly requires an amine co-initiator, which may contribute to colour changes when residual components remain after polymerisation [18,19]. Alternative photoinitiators such as 1-phenyl-1,2-propanedione (PPD), bis(acyl)phosphine oxide (BAPO), and diphenyl(2,4,6-trimethylbenzoyl) phosphine oxide (TPO) have therefore been investigated. Their different absorption spectra, photobleaching behaviour, and polymerisation mechanisms can influence the resulting optical properties of the composite [18,19,20]. Consequently, comparing different photoinitiator systems within the same RHS-containing resin matrix may help clarify their contribution to early post-polymerisation colour change.

Colour change is commonly quantified using the CIELAB system, in which L*, a*, and b* describe lightness and the red–green and yellow–blue axes, respectively, and ΔE represents the overall colour difference [6,21]. However, recorded ΔE values can also be influenced by the measurement method, including the instrument, background, specimen thickness, illumination geometry, and calibration procedure [3,21,22,23]. Different colour-assessment devices may therefore produce different values for the same material. For this reason, both material formulation and measurement device should be considered when evaluating post-polymerisation colour change.

Since the aesthetic performance of restorative materials depends on both composition and measurement protocol, it is necessary to evaluate different RHS-incorporated FCs using more than one colour-measuring device. Therefore, the present study aimed to compare the 24 h post-polymerisation colour change in experimental RHS-incorporated FCs containing CQ, PPD, CQ/PPD, BAPO, and TPO photoinitiator systems, with two commercial composites included as reference materials, using VITA Easyshade, a Ds-700d portable spectrophotometer, and ColorMeter Pro. The null hypothesis was that measurement device and composite material would not significantly influence ΔE*ab values and that there would be no device × composite interactions.

## 2. Materials and Methods

The experimental flowable composites (FCs) were prepared using two RHS-incorporated resin formulations, designated U50 and U40. Both formulations contained 50 wt% nanohybrid RHS filler and 50 wt% resin matrix composed of urethane dimethacrylate (UDMA) and triethylene glycol dimethacrylate (TEGDMA). In U50, UDMA and TEGDMA were mixed at a 50:50 ratio, whereas U40 contained a 40:60 UDMA/TEGDMA ratio. Each resin formulation was further divided into five photoinitiator groups: CQ, PPD, CQ/PPD, BAPO, and TPO. CQ and PPD were each used in combination with the amine co-initiator 2-(dimethylamino)ethyl methacrylate (DMAEMA), whereas CQ/PPD, BAPO, and TPO were formulated without DMAEMA. All experimental materials were protected from ambient light until specimen fabrication. Two commercial flowable composites, Filtek Supreme Ultra Flowable Restorative (3M ESPE, St. Paul, MN, USA) and Revolution Formula 2 flowable light cure composite (Kerr Corporation, Brea, CA, USA), were included as reference materials. The compositions and group designations of the experimental FCs are summarized in Table 1.

Sample size was determined a priori using G*Power version 3.1.9. Based on an effect size of 0.779 [24], α = 0.05, and 80% power, the minimum required sample size was four specimens per group. For the cross-device repeated-measures analysis, five matched specimens from each of the 12 composite groups (10 experimental and two commercial reference groups) were included, yielding a total sample of *N* = 60. Only specimens with complete measurements from all three devices were analyzed. Although five specimens per group represents a small sample size, this exceeded the a priori requirement and is consistent with previous in vitro colour research using five specimens per material subgroup [24,25].

**Figure 1:**
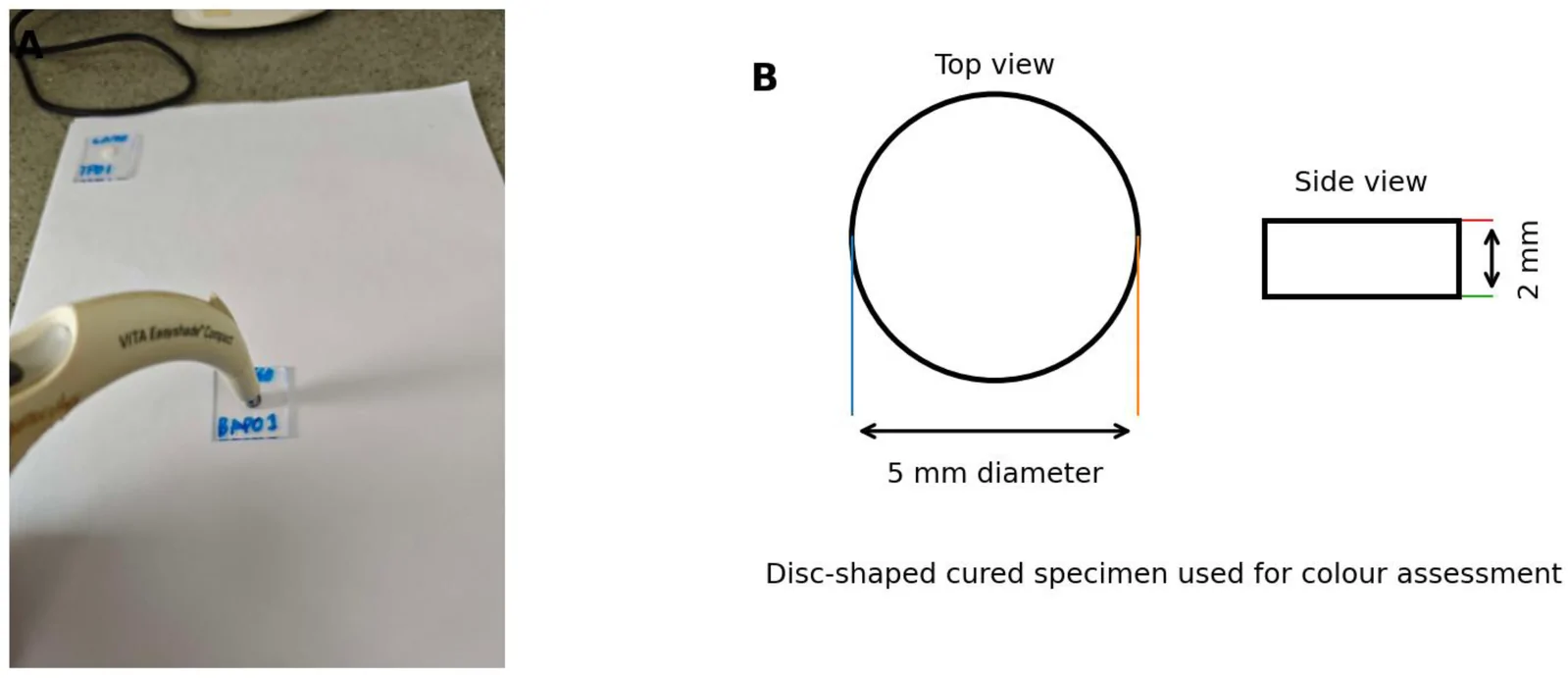
Representative specimen and measurement geometry. (**A**) Laboratory photograph showing a cured experimental composite specimen positioned on the standardized white backing during VITA Easyshade measurement. (**B**) Schematic of the disc-shaped specimen used for colour assessment (5 mm diameter × 2 mm thickness).

Disc-shaped specimens measuring 5 mm in diameter and 2 mm in thickness were fabricated in acrylic moulds (Figure 1) between mylar strips and glass slides. The composite was inserted carefully to minimize air entrapment, and the upper surface was flattened with a mylar strip and glass slide. Specimens were polymerised from the top surface for 40 s using an Elipar DeepCure LED light-curing unit (3M ESPE, USA) at an irradiance of approximately 1200 mW/cm^2^ measured with a radiometer. The curing tip was positioned perpendicular to the centre of the specimen surface (Figure 1). A 2 mm thickness was selected to provide a standardized optical path and to remain consistent with common laboratory resin-composite colour protocols and conventional incremental placement; Yu and Lee likewise used 2 mm-thick specimens [25]. Thinner clinically relevant layers (e.g., 1.0 or 1.5 mm) were not included in the present design and are considered in the discussion because composite thickness can alter L*, a*, b*, translucency, and background contribution [26].

Immediately after curing, CIELAB L*, a*, and b* coordinates [27,28] were recorded at the centre of each specimen using VITA Easyshade Compact (VITA Zahnfabrik, Bad Säckingen, Germany), designated D1; DS-700D portable spectrophotometer (CHN Spec Technology, Hangzhou, China), designated D2; and ColorMeter Pro (CHN Spec Technology, China), designated D3. The specimens were then stored dry and in darkness at 37 °C for 24 h, after which measurements were repeated at the same location and orientation. All measurements were performed by the same operator under consistent laboratory lighting and temperature, with each specimen positioned on a white A4-paper background. Each instrument was calibrated according to the manufacturer’s instructions before each measurement session. Multiple readings were obtained from the central area of each specimen at each time point and averaged to generate the final L*, a*, and b* values used for ΔE calculation. Although the same white background and measurement conditions were used throughout the study, the background was not a calibrated optical standard. In addition, device-specific aperture and measurement-mode settings were not available in the archived records, which may limit reproducibility and comparison with studies using different measurement protocols. These were acknowledged as reporting limitations.

Total colour change was calculated using the CIELAB [27,28] colour-difference equation ΔE*ab = [(ΔL*)^2^ + (Δa*)^2^ + (Δb*)^2^]^1^/^2^, where ΔL*, Δa*, and Δb* represent the differences between the immediate post-cure and 24 h measurements.

Colour change was measured on the same specimens using D1, D2, and D3; therefore, device-specific ΔE*ab values were treated as repeated measurements. The analysis included 12 composite groups with five specimens per group (N = 60). A mixed repeated-measures ANOVA was performed in SPSS version 29, with device (D1, D2, D3) (three levels) as the within-subject factor and composite (12 levels) as the between-subject factor. Sphericity and homogeneity assumptions were assessed using Mauchly’s test, Box’s M test, and Levene’s test. Bonferroni-adjusted comparisons were used for follow-up analyses, with statistical significance set at *p* < 0.05 and partial η^2^ reported as the effect-size estimate.

Agreement among devices was assessed separately using pairwise Bland–Altman analyses [29,30] and a two-way mixed-effects, single-measurement, absolute-agreement intraclass correlation coefficient (ICC). Bias and 95% limits of agreement were calculated for D1–D2, D1–D3, and D2–D3, with 10,000 stratified bootstrap resamples used to obtain confidence intervals. Proportional bias was assessed by linear regression. Bland–Altman and ICC analyses were performed in Python version 3.13.5 with NumPy 2.3.5, pandas 2.2.3, SciPy 1.17.0, and Matplotlib 3.10.8. used for data handling, statistical calculations, regression analysis, and graphical presentation.

## 3. Results

**Figure 2:**
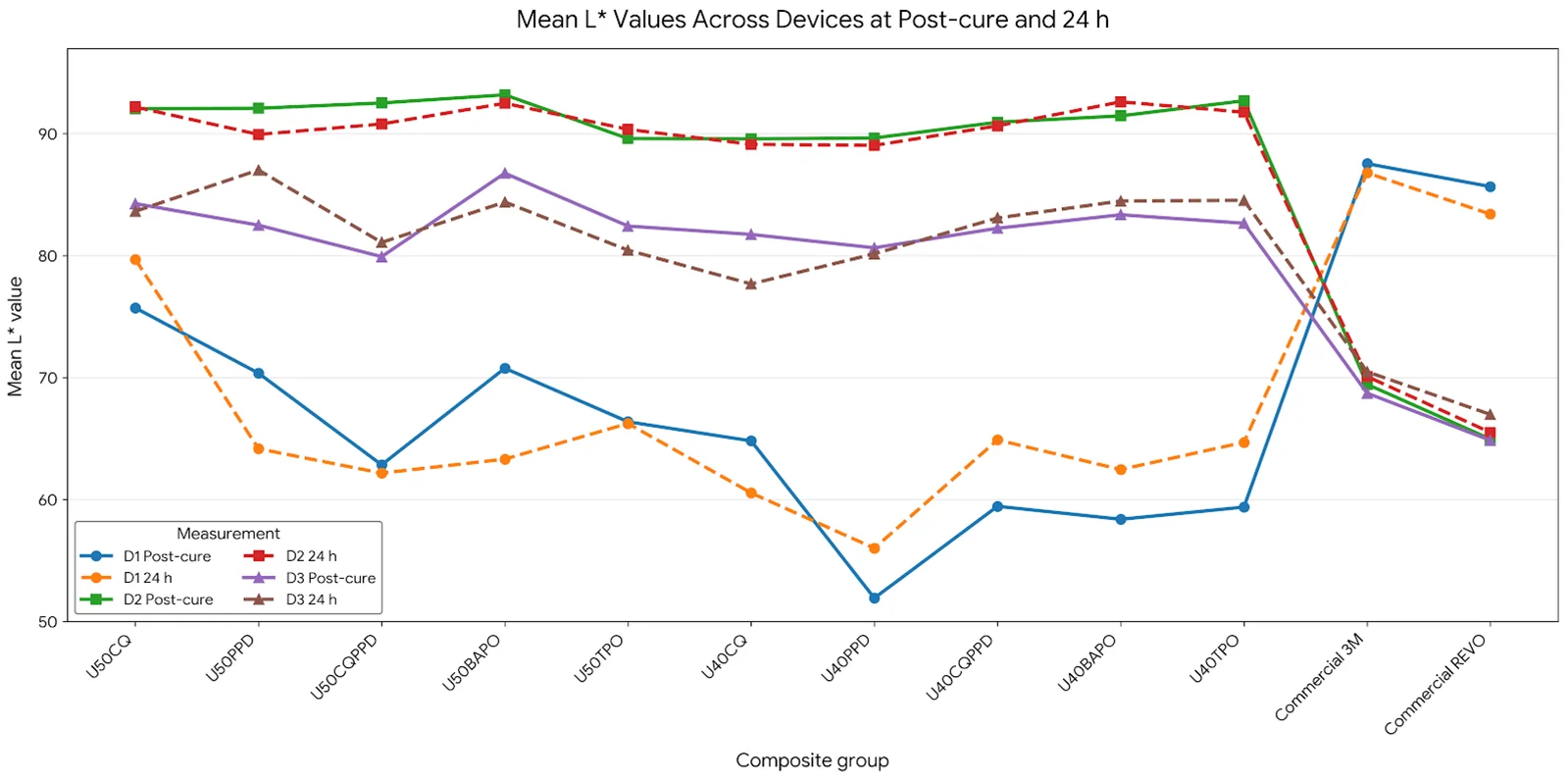
Mean L* values measured by D1, D2, and D3 immediately post-cure and after 24 h.

**Figure 3:**
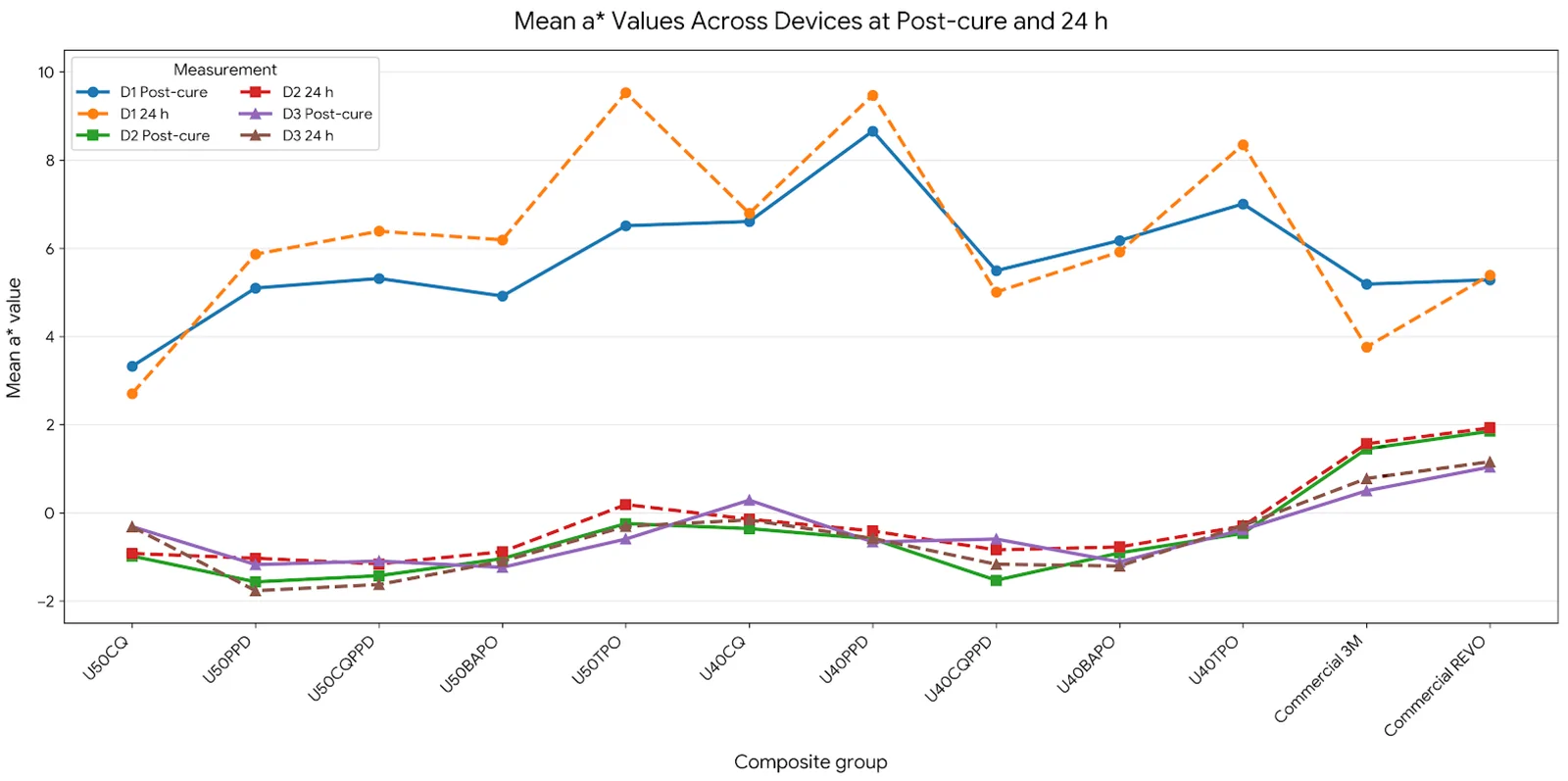
Mean a* values measured by D1, D2, and D3 immediately post-cure and after 24 h.

**Figure 4:**
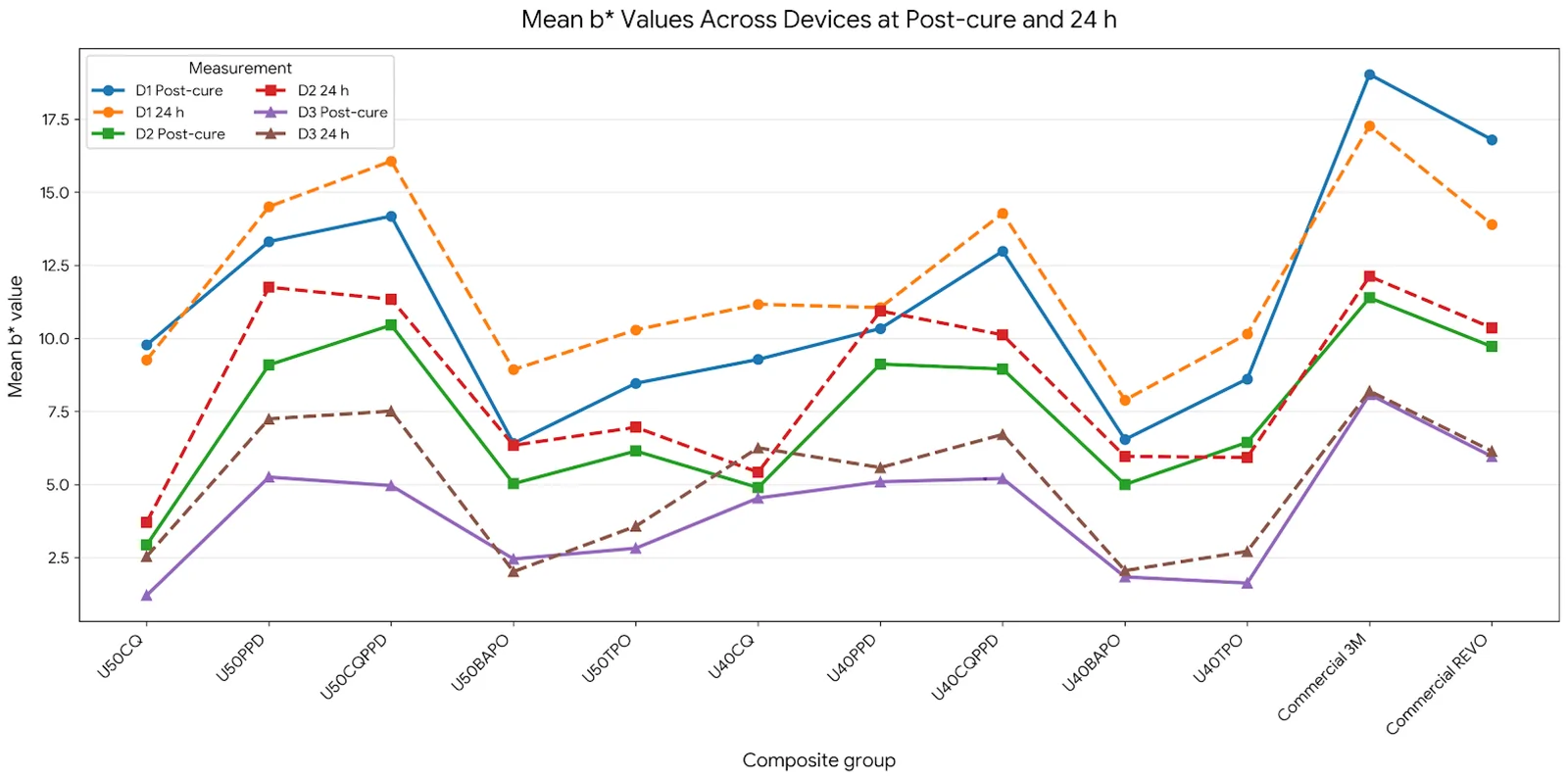
Mean b* values measured by D1, D2, and D3 immediately post-cure and after 24 h.

The coordinate-level changes are presented first in Figure 2, Figure 3 and Figure 4. Figure 2 shows clear device-dependent separation in L*. For the experimental groups, D2 generally recorded the highest lightness values, D1 the lowest, and D3 intermediate values; however, the commercial materials showed a different device ranking. The direction and magnitude of the 24 h change also varied by group, indicating that the recorded lightness shift reflected a device-material response rather than uniform darkening or brightening.

Figure 3 demonstrates that the a* coordinate was particularly sensitive to the measuring device. D1 produced predominantly positive a* values for the experimental groups, whereas D2 and D3 generally recorded negative values, indicating different placement on the red–green axis. The post-cure-to-24 h shifts were group dependent and were generally smaller for D2 and D3 than for D1.

Figure 4 shows positive b* values for all materials, indicating a measurable yellow component, but the magnitude differed considerably among devices. D1 generally recorded the highest b* values, D3 the lowest, and D2 intermediate values. PPD- and CQ/PPD-containing groups and Commercial 3M showed relatively high b* values, while the 24 h changes were group dependent, supporting the view that yellowing or photobleaching cannot be interpreted independently of the instrument used.

For the repeated-measures ANOVA, Mauchly’s test was not significant (W = 0.965, *p* = 0.432), indicating that the sphericity assumption was satisfied. There was a significant main effect of device, F(2, 96) = 251.81, *p* < 0.001, partial η^2^ = 0.840, and a significant main effect of composite, F(11, 48) = 14.54, *p* < 0.001, partial η^2^ = 0.769. The device × composite interaction was also significant, F(22, 96) = 5.22, *p* < 0.001, partial η^2^ = 0.545 (Table 2), indicating that the magnitude of the device differences depended on the composite material.

**Figure 5:**
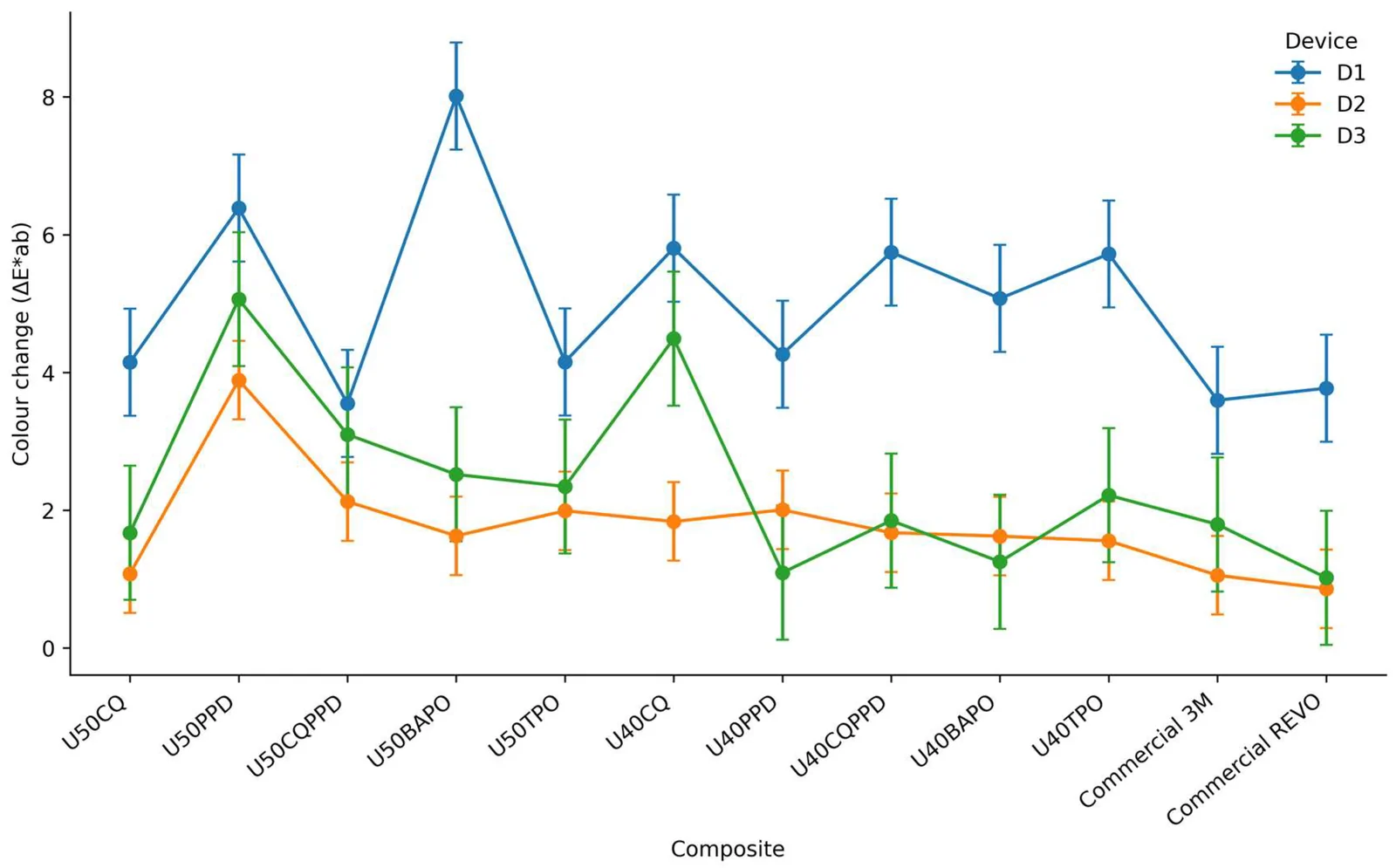
Device × composite interaction for the original ΔE*ab data. Points represent device-specific means and error bars represent model-based 95% confidence intervals.

Across all composites (Table 3 and Figure 5), the estimated mean ΔE*ab was highest for D1 (5.019), followed by D3 (2.368) and D2 (1.777). Bonferroni-adjusted pairwise comparisons showed significant differences between D1 and D2 (mean difference = 3.242, *p* < 0.001), D1 and D3 (mean difference = 2.651, *p* < 0.001), and D2 and D3 (mean difference = −0.591, *p* = 0.002).

**Figure 6:**
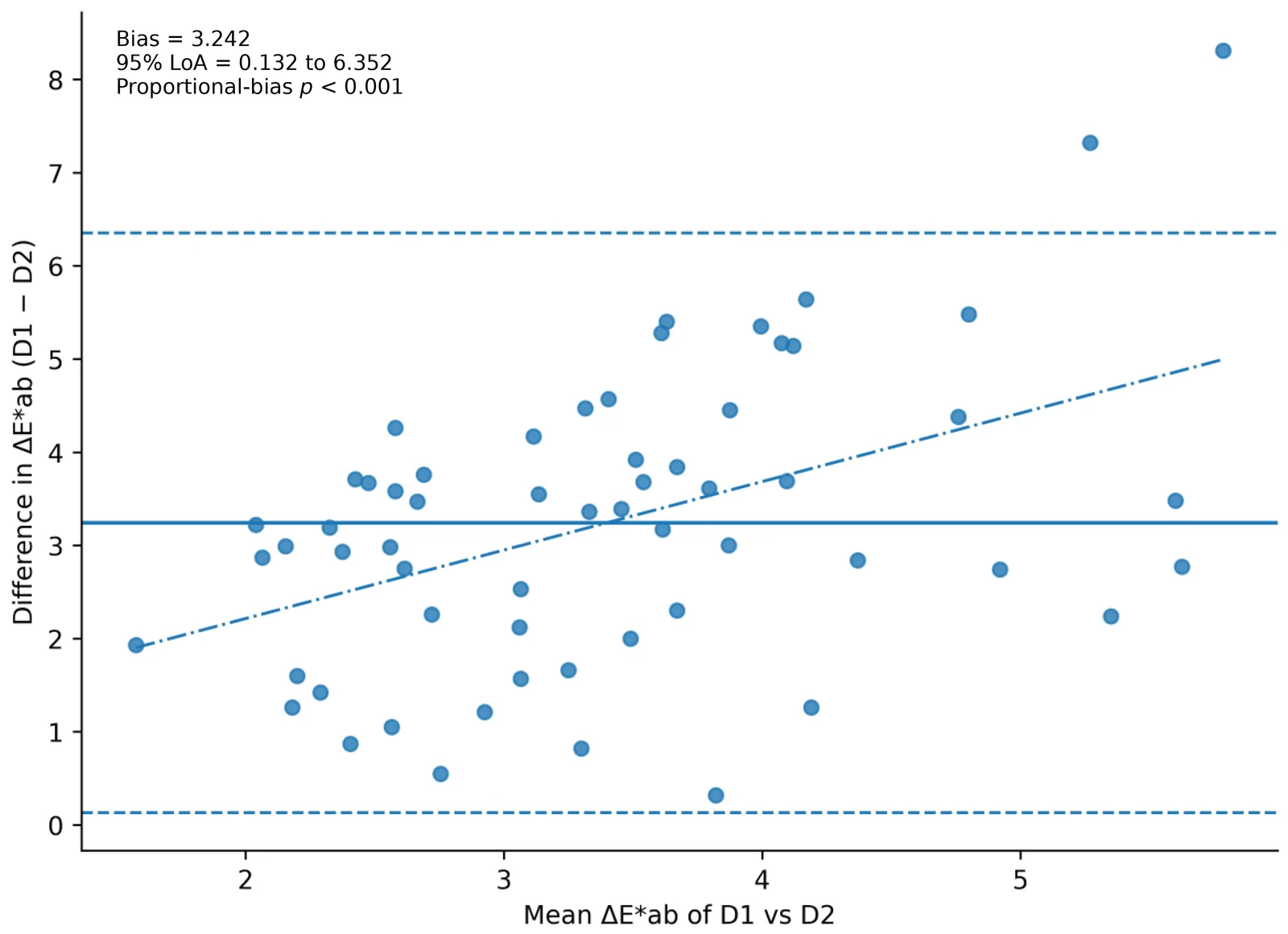
Bland–Altman plot for D1 versus D2. The solid horizontal line denotes mean bias and dashed lines denote the 95% limits of agreement. The dash-dot line shows the significant proportional bias regression.

**Figure 7:**
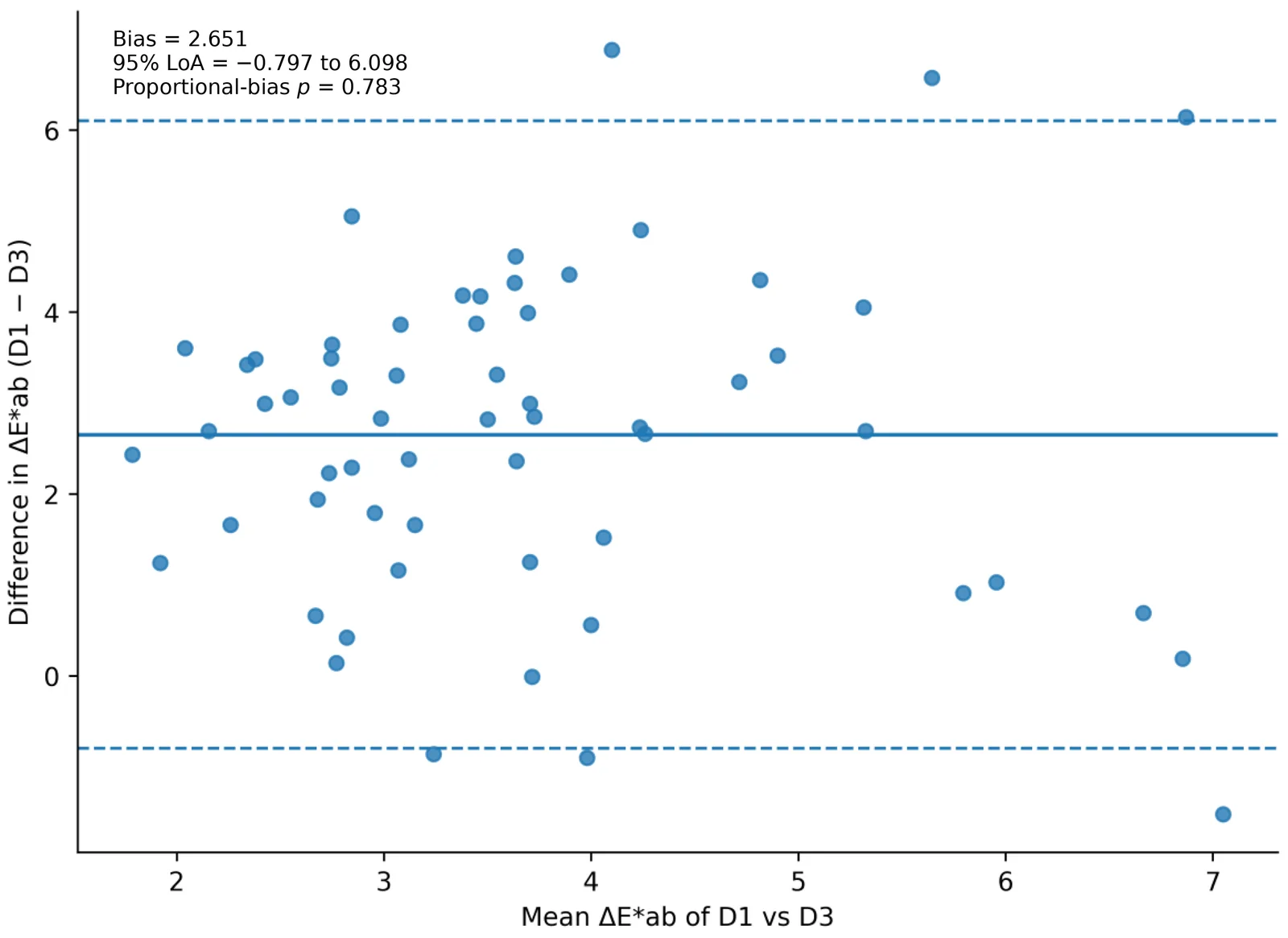
Bland–Altman plot for D1 versus D3. The solid horizontal line denotes mean bias and dashed lines denote the 95% limits of agreement.

**Figure 8:**
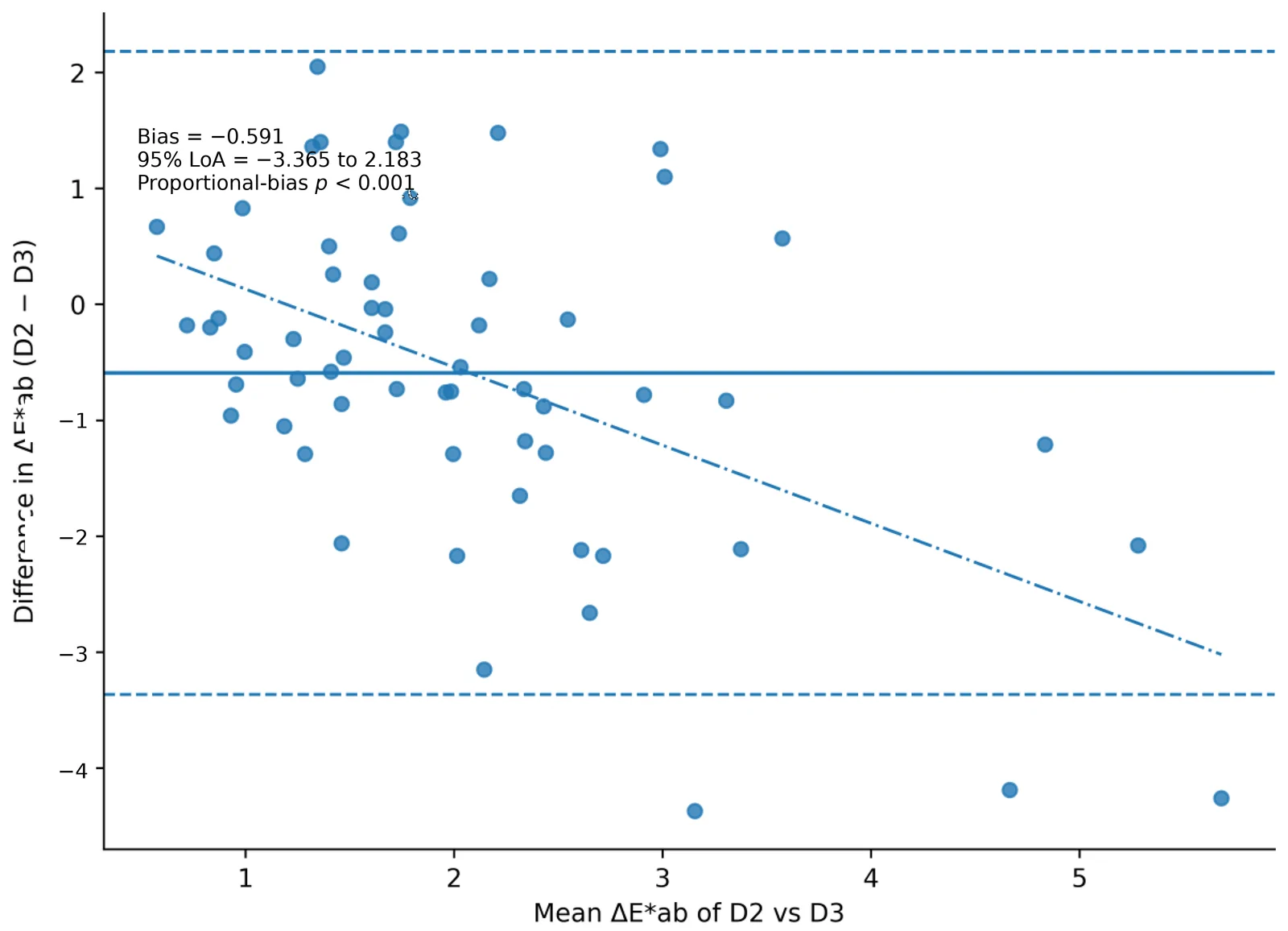
Bland–Altman plot for D2 versus D3. The solid horizontal line denotes mean bias and dashed lines denote the 95% limits of agreement. The dash-dot line shows the significant proportional bias regression.

For the agreement analysis (Table 4 and Figure 6, Figure 7 and Figure 8), for D1 versus D2, Bland–Altman analysis showed a mean bias of 3.242 ΔE*ab, with 95% LoA from 0.132 to 6.352. A significant proportional bias was detected (slope = 0.735, *p* < 0.001). For D1 versus D3, the mean bias was 2.651 ΔE*ab, with 95% LoA from −0.797 to 6.098; proportional bias was not significant (*p* = 0.783). For D2 versus D3, the mean bias was -0.591 ΔE*ab, with 95% LoA from −3.365 to 2.183, and significant proportional bias was present (slope = −0.673, *p* < 0.001). The two-way mixed-effects, single-measurement, absolute-agreement ICC across D1, D2 and D3 was 0.130 (stratified bootstrap 95% CI 0.078–0.177), indicating poor absolute agreement.

## 4. Discussion

The null hypothesis was rejected because composite formulation and measurement device and their interactions significantly influenced ΔE. The repeated-measures results show that the three devices produced systematically different estimates of colour change, and the significant device × composite interaction indicates that the magnitude of these differences was material dependent. This is consistent with the dental colour-measurement literature showing that spectrophotometric readings may vary across instruments because of differences in optical geometry, measurement area, illumination, detector configuration and internal processing [31,32]. Previous studies have likewise reported that colour values obtained from different dental spectrophotometers are not necessarily directly interchangeable, even when repeatability within a device is acceptable [33,34,35,36].

The coordinate-level plots (Figure 2, Figure 3 and Figure 4) show clear device-dependent differences in the recorded CIELAB coordinates [27,28]. D2 generally placed the experimental specimens at higher L* values than D1, while the a* polarity and b* magnitudes also differed among devices. Because ΔE*ab is calculated from changes in L*, a*, and b*, even modest device-specific differences in one coordinate can alter the total colour-difference value. Differences in aperture size, illumination, detector geometry, calibration, measurement mode, surface reflection, and software processing therefore remain plausible explanations for the distinct device responses [21,23,31,32].

The agreement analyses strengthen this interpretation because they quantify the size and pattern of disagreement rather than only testing whether mean values differ. D1 showed a positive systematic bias relative to both D2 (3.242 ΔE*ab) and D3 (2.651 ΔE*ab), confirming that D1 generally reported greater colour change. The D2–D3 bias was smaller (−0.591 ΔE*ab), but the limits of agreement remained wide. In addition, significant proportional bias for D1–D2 and D2–D3 indicates that the difference between those devices changes according to the magnitude of the measured colour change; consequently, a single constant correction factor would not adequately reconcile those device pairs across the full measurement range [29,30].

The absolute-agreement ICC of 0.130 was low. According to commonly used ICC interpretation guidance, values below 0.50 indicate poor reliability/agreement [37]. Taken together, the ANOVA, Bland–Altman plots and ICC therefore do not support treating D1, D2 and D3 as interchangeable measurement devices in the present dataset. It is important, however, to distinguish statistical agreement from clinical acceptability: Bland–Altman analysis describes bias and limits of agreement, but a formal claim of clinical interchangeability requires an acceptability margin defined on scientific or clinical grounds rather than selected retrospectively from the observed data [29,30]. Dental perceptibility and acceptability thresholds may help contextualize colour differences, but they should not automatically be treated as equivalence limits for differences between two devices’ ΔE measurements [38].

The significant device × composite interaction also supports a material-dependent optical response. Photoinitiator chemistry can influence residual colour, photobleaching and polymerisation, while the UDMA/TEGDMA ratio can modify network formation, residual monomer content and refractive-index relationships. CQ is intrinsically yellow and is used with an amine co-initiator; oxidation of residual amine or incomplete photobleaching may contribute to colour change [39]. Conversely, TPO and BAPO are amine-free Type I photoinitiators. These mechanisms may affect L*, a* and particularly b* to different extents, and a device that is more sensitive to a given coordinate or surface-reflection condition may therefore record a different magnitude of ΔE*ab for the same material [18,19,21,23]. Mechanistically, CQ/amine, PPD/amine, CQ/PPD, BAPO, and TPO can differ in intrinsic colour, spectral absorption, photobleaching kinetics, radical-generation pathways, and the amount or type of residual initiator/co-initiator after curing. These differences may influence conversion, network formation, refractive-index matching, translucency, and light scattering, thereby altering the recorded L*, a*, and particularly b* coordinates. Because the three devices differ in illumination/detector geometry, sampling area, calibration, and signal processing, the same photoinitiator-dependent optical change may be weighted differently by each instrument [18,19,21,23]. In the present analysis, the significant device × composite interaction supports a material-dependent device response; however, it should not be interpreted as a separately tested photoinitiator × device interaction because the between-subject factor was the 12-level composite factor.

The short 24 h evaluation period should also be considered when interpreting these findings. Since specimens were stored dry and in darkness, the measured changes primarily characterize early post-polymerisation colour change, including photoinitiator bleaching and stabilization of the polymer network, rather than long-term staining or degradation in the oral environment. Longer ageing periods and immersion in standardized staining or pH-controlled media are required to determine whether the same material- and device-related patterns persist under more clinically relevant conditions [4,8,9,10,11,40,41,42,43]. Accordingly, the endpoint in this study is more accurately interpreted as early post-polymerisation colour change and photobleaching rather than long-term clinical colour stability.

The curing-related literature is also closely linked to the broader experimental findings because different photoinitiator systems and polymerisation efficiency were one of the principal variables distinguishing the experimental groups. Curing duration and the original composite shade can alter radiant-energy transmission, conversion, residual initiator concentration, and baseline optical coordinates, thereby changing the amount of colour difference recorded during ageing [44]. Thus, the photoinitiator may play a role in the colour change values among the groups together with other factors mentioned previously.

Specimen thickness is an additional limitation. A single 2 mm thickness was used to standardize the optical path and to match a commonly used laboratory thickness, but 1.0 and 1.5 mm layers can also be clinically relevant. Because resin-composite thickness influences CIELAB coordinates, translucency, and the contribution of the background, the absolute L*, a*, b*, and ΔE*ab values observed here should not be assumed to apply unchanged to thinner restorations [26]. Future studies should directly compare multiple clinically relevant thicknesses using the same calibrated device and background.

The measurement protocol itself is another limitation. The use of a white A4-paper background, the selected 2 mm specimen thickness, instrument-specific aperture and geometry, and potential differences in sensitivity among the three systems can influence measurements of translucent composites. Future studies should standardize the device, calibration method, background, ambient lighting, number of repeated readings, measurement location, surface-finishing procedure, specimen thickness, and measurement geometry before comparing colour change across materials or instruments [6,21]. The white A4 paper was a practical, consistent high-reflectance backing rather than a calibrated optical reference, and this may affect measurements of translucent specimens. Moreover, the archived records do not retain the exact replicate-reading count or all instrument-specific aperture and measurement-mode settings. These reporting constraints limit reproducibility. More advanced dental or laboratory colour-measurement systems may provide tighter control of measurement geometry or more comprehensive spectral and shade information; consequently, the present device-specific findings should not be generalized to all colour-assessment systems [21,23].

The study should also be interpreted with consideration of the small number of specimens per composite (*n* = 5) and the previously observed heterogeneity of variance for D3. Future work with a larger number of specimens and replicate readings from each instrument would allow device-specific repeatability, within-device precision and more advanced agreement models to be estimated. Nevertheless, the matched *n* = 5 per composite exceeded the a priori power requirement and is comparable with published in vitro colour research using five specimens per material subgroup [24,25].

## 5. Conclusions

Within the limitations of this study, D1, D2 and D3 produced significantly different assessments of 24 h post-polymerisation colour change, and the size of the differences varied among composite materials. Bland–Altman analysis demonstrated systematic positive bias for D1 relative to D2 and D3, with proportional bias for D1–D2 and D2–D3. The overall absolute-agreement ICC was poor. These findings indicate that the three devices should not be assumed to be directly interchangeable for ΔE*ab assessment. As specimens were stored dry and in darkness for only 24 h, the results should be interpreted as early post-polymerisation colour change/photobleaching rather than long-term clinical colour stability. For longitudinal colour assessments, using the same calibrated device throughout a given comparison under standardized measurement conditions is therefore advisable.

## Figures and Tables

**Table 1:** Composition and group designation of the experimental RHS-incorporated and commercial flowable composites.

Group	Resin Matrix Ratio	Filler Loading	Photoinitiator/Co-Initiator Composition (g)
U50CQ	50:50 (UDMA: TEGDMA)	50 wt.% RHS	CQ 0.02 g + DMAEMA 0.01 g
U50PPD	50:50 (UDMA: TEGDMA)	50 wt.% RHS	PPD 0.02 g + DMAEMA 0.01 g
U50CQPPD	50:50 (UDMA: TEGDMA)	50 wt.% RHS	CQ 0.02 g + PPD 0.02 g
U50BAPO	50:50 (UDMA: TEGDMA)	50 wt.% RHS	BAPO 0.02 g
U50TPO	50:50 (UDMA: TEGDMA)	50 wt.% RHS	TPO 0.02 g
U40CQ	40:60 (UDMA: TEGDMA)	50 wt.% RHS	CQ 0.02 g + DMAEMA 0.01 g
U40PPD	40:60 (UDMA: TEGDMA)	50 wt.% RHS	PPD 0.02 g + DMAEMA 0.01 g
U40CQPPD	40:60 (UDMA: TEGDMA)	50 wt.% RHS	CQ 0.02 g + PPD 0.02 g
U40BAPO	40:60 (UDMA: TEGDMA)	50 wt.% RHS	BAPO 0.02 g
U40TPO	40:60 (UDMA: TEGDMA)	50 wt.% RHS	TPO 0.02 g
Revolution Formula 2 (Revo)	Bis-GMA, TEGDMA	60 wt.%, 0.6 µm proprietary glass filler	Not disclosed by the company. Reported to be CQ-based system
Filtek Supreme Ultra Restorative (Filtek 3M)	Bis-GMA, Bis-EMA, UDMA, and PEGDMA/TEGDMA	65 wt.%, Silica, zirconia/silica clusters and ytterbium trifluoride	CQ-based system

All experimental formulations contained 50 wt.% nanohybrid RHS filler and 50 wt.% UDMA/TEGDMA resin matrix. RHS = rice husk silica; UDMA = urethane dimethacrylate; TEGDMA = triethylene glycol dimethacrylate; CQ = camphorquinone; PPD = 1-phenyl-1,2-propanedione; BAPO = bis(acyl)phosphine oxide; TPO = diphenyl(2,4,6-trimethylbenzoyl)phosphine oxide; DMAEMA = 2-(dimethylamino)ethyl methacrylate.

**Table 2:** Mixed repeated-measures ANOVA of ΔE*ab according to measurement device and composite material.

Effect	df	F	*p*	Partial η^2^
Device	2, 96	251.812	<0.001	0.840
Composite	11, 48	14.538	<0.001	0.769
Device × Composite	22, 96	5.219	<0.001	0.545

Mauchly’s test indicated that sphericity was satisfied (W = 0.965, *p* = 0.432); therefore, sphericity-assumed degrees of freedom are reported.

**Table 3:** Colour change (ΔE*ab) measured by D1, D2 and D3 for each composite material.

Composite	D1, Mean ± SD	D2, Mean ± SD	D3, Mean ± SD
U50CQ	4.15 ± 1.00 ^a^	1.08 ± 0.70 ^b^	1.67 ± 0.70 ^b^
U50PPD	6.39 ± 0.97 ^a^	3.89 ± 0.34 ^b^	5.06 ± 2.19 ^ab^
U50CQPPD	3.55 ± 0.43 ^a^	2.13 ± 0.56 ^b^	3.10 ± 1.20 ^ab^
U50BAPO	8.01 ± 1.38 ^a^	1.63 ± 0.27 ^b^	2.52 ± 1.17 ^b^
U50TPO	4.15 ± 0.90 ^a^	1.99 ± 1.21 ^b^	2.34 ± 0.21 ^b^
U40CQ	5.80 ± 1.16 ^a^	1.84 ± 0.71 ^b^	4.49 ± 1.54 ^a^
U40PPD	4.27 ± 0.89 ^a^	2.01 ± 0.56 ^b^	1.09 ± 0.32 ^b^
U40CQPPD	5.75 ± 0.85 ^a^	1.67 ± 0.50 ^b^	1.85 ± 0.60 ^b^
U40BAPO	5.07 ± 0.43 ^a^	1.62 ± 0.56 ^b^	1.25 ± 0.70 ^b^
U40TPO	5.72 ± 0.41 ^a^	1.56 ± 0.84 ^b^	2.22 ± 0.75 ^b^
Commercial 3M Filtek	3.60 ± 0.59 ^a^	1.06 ± 0.44 ^b^	1.79 ± 1.31 ^b^
Commercial Revo	3.77 ± 0.75 ^a^	0.86 ± 0.24 ^b^	1.02 ± 0.54 ^b^

Within each row, values sharing at least one superscript letter are not significantly different. Values with no superscript letter in common differ significantly by Bonferroni-adjusted simple-main-effect comparisons of device (*p* < 0.05). *n* = 5 per composite.

**Table 4:** Bland–Altman agreement statistics for pairwise comparison of the three devices.

Comparison	Bias (95% CI)	Lower LoA (95% Bootstrap CI)	Upper LoA (95% Bootstrap CI)	Proportional Bias Slope	*p*
D1 − D2	3.242 (2.832 to 3.652)	0.132 (−0.361 to 0.684)	6.352 (5.786 to 6.895)	0.735	<0.001
D1 − D3	2.651 (2.196 to 3.105)	−0.797 (−1.387 to −0.129)	6.098 (5.556 to 6.554)	−0.050	0.783
D2 − D3	−0.591 (−0.957 to −0.226)	−3.365 (−3.955 to −2.696)	2.183 (1.680 to 2.615)	−0.673	<0.001

LoA = 95% limit of agreement. Bootstrap confidence intervals are based on 10,000 stratified resamples. The two-way mixed-effects, single-measurement, absolute-agreement ICC across D1, D2 and D3 was 0.130 (bootstrap 95% CI 0.078–0.177).

## Data Availability

The original contributions presented in this study are included in the article. Further inquiries can be directed to the corresponding authors.

## Abbreviations

The following abbreviations are used in this manuscript:

ANOVA	Analysis of variance
BAPO	Bis-acylphosphine oxide
CIELAB	Commission Internationale de l’Éclairage L*a*b* colour space
CQ	Camphorquinone
D1	VITA Easyshade
D2	Ds-700d Portable Spectrophotometer
D3	ColorMeter Pro
DMAEMA	2-(Dimethylamino)ethyl methacrylate
ΔE	Total colour difference
LED	Light-emitting diode
PPD	Phenylpropanedione
RHS	Rice husk silica
SD	Standard deviation
SPSS	Statistical Package for the Social Sciences
TEGDMA	Triethylene glycol dimethacrylate
TPO	Diphenyl(2,4,6-trimethylbenzoyl)phosphine oxide
U40	Experimental formulation containing UDMA and TEGDMA in a 40:60 ratio
U50	Experimental formulation containing UDMA and TEGDMA in a 50:50 ratio
UDMA	Urethane dimethacrylate
ICC	Intraclass correlation coefficient
LoA	Limit of agreement
